# Pulse of Animals

**Published:** 1874-01

**Authors:** 


Pulse of Various Animals. — The pulse of our domestic animals, as given by Vatel, in his Veterinary Pathology, is as follows :—Horse, from 32 to 38 pulsations per minute ; ox or cow, 25 to 42 ; ass, 48 to 54 ; sheep, 70 to 79 ; dog, 90 to 100 ; cat, 110 to 120 ; rabbit, 120 ; guinea pig, 140 ; duck, 135 ; hen, 140.

